# Voluntary Medical Male Circumcision Scale-Up in Nyanza, Kenya: Evaluating Technical Efficiency and Productivity of Service Delivery

**DOI:** 10.1371/journal.pone.0118152

**Published:** 2015-02-23

**Authors:** Dickens S. Omondi Aduda, Collins Ouma, Rosebella Onyango, Mathews Onyango, Jane Bertrand

**Affiliations:** 1 Department of Public Health and Community Development, Maseno University, Maseno, Kenya; 2 Department of Biomedical Sciences and Technology, Maseno University, Maseno, Kenya; 3 Male Circumcision Consortium, FHI360, Kisumu Office, Kisumu, Kenya; 4 Department of Global Health Systems and Development, Tulane University. New Orleans, LA, 70112, United States of America; Johns Hopkins Bloomberg School of Public Health, UNITED STATES

## Abstract

**Background:**

Voluntary medical male circumcision (VMMC) service delivery is complex and resource-intensive. In Kenya’s context there is still paucity of information on resource use vis-à-vis outputs as programs scale up. Knowledge of technical efficiency, productivity and potential sources of constraints is desirable to improve decision-making.

**Objective:**

To evaluate technical efficiency and productivity of VMMC service delivery in Nyanza in 2011/2012 using data envelopment analysis.

**Design:**

Comparative process evaluation of facilities providing VMMC in Nyanza in 2011/2012 using output orientated data envelopment analysis.

**Results:**

Twenty one facilities were evaluated. Only 1 of 7 variables considered (total elapsed operation time) significantly improved from 32.8 minutes (SD 8.8) in 2011 to 30 minutes (SD 6.6) in 2012 (95%CI = 0.0350–5.2488; *p* = 0.047). Mean scale technical efficiency significantly improved from 91% (SD 19.8) in 2011 to 99% (SD 4.0) in 2012 particularly among outreach compared to fixed service delivery facilities (CI -31.47959–4.698508; *p* = 0.005). Increase in mean VRS technical efficiency from 84% (SD 25.3) in 2011 and 89% (SD 25.1) in 2012 was not statistically significant. Benchmark facilities were #119 and #125 in 2011 and #103 in 2012. Malmquist Productivity Index (MPI) at fixed facilities declined by 2.5% but gained by 4.9% at outreach ones by 2012. Total factor productivity improved by 83% (p = 0.032) in 2012, largely due to progress in technological efficiency by 79% (p = 0.008).

**Conclusions:**

Significant improvement in scale technical efficiency among outreach facilities in 2012 was attributable to accelerated activities. However, ongoing pure technical inefficiency requires concerted attention. Technological progress was the key driver of service productivity growth in Nyanza. Incorporating service-quality dimensions and using stepwise-multiple criteria in performance evaluation enhances comprehensiveness and validity. These findings highlight site-level resource use and sources of variations in VMMC service productivity, which are important for program planning.

## Introduction

Service delivery of VMMC for HIV intervention was rolled out in Kenya in 2008. The program *(i)* is characterized by a complex and resource intensive delivery function, which has considerable implications for both technical and functional program outcomes [1,2]; *(ii)* efficiency and productivity portends the program’s impact on HIV epidemic and policy directions [3]; *(iii)* require wide and rapid coverage to realize the intended public health impact [2,4]; *(iv)* resource allocation and use require objective information on both institutional and micro-level service delivery performance to enhance decision-making.[1] Hitherto, most studies on VMMC services have focused on program cost-effectiveness [4,5,6,7] and how it works [8,9,10,11]. The current study builds on existing knowledge of how the program works by examining the technical efficiency and productivity dimensions of service delivery in Nyanza region, Kenya, to determine the extent of resource use by service facilities vis-à-vis selected outputs. The study results are critical to augmenting VMMC service delivery management solutions.

Service delivery is the key function of the health systems, and it is defined as ‘the way inputs are combined to allow provision of a series of interventions or health actions’ to promote, restore or maintain health in an equitable manner.[12] The prevalent perspectives for evaluating service delivery consider: *(i)* the relationship between inputs (such as manpower and capital) available for service delivery and the outputs (including services, products, or technologies) that results from health care activities (productivity perspective); and *(ii)* performance of service delivery in terms of the health effects or status change resulting from the outputs (effectiveness perspective).[13] Technical efficiency and productivity of voluntary medical male circumcision (VMMC) services was evaluated based on the first perspective.

Technical efficiency measures the ability of a facility to produce the maximum quantity of program outputs for any given amount of inputs or the minimum input levels used for any given amount of outputs. Service productivity identifies ‘the change in service output resulting from a unit change in the inputs’ over time.[14] Service quality dimensions were considered central to service delivery function hence a key variable in identifying benchmark units (ideal performance units set on the basis of a sample of similar facilities and performance over time).[15,16] The conceptual framework for evaluating these measures encompasses: *i) inputs* (clinicians, nurses, surgical bed, surgical time); *ii) process* (structure such as tasks performed during circumcision) and; *iii) output* (services including number of circumcisions accomplished, proportion of circumcised men receiving HIV test, service quality).[17]

### Efficiency, benchmarking and productivity evaluation of VMMC service delivery

Evaluation of a service delivery plan for VMMC involves several dimensions including inputs used, outputs generated and service quality.[18] Simultaneous consideration of multiple dimensions of service delivery accords a platform to demonstrate how resources are used in diverse contexts (in terms of input-output mix) among different producing units. Evaluation indicators would normally be designed in relation to one or multiple dimensions selected.[19,20] When multiple dimensions are observed, composite indicators (defined as a combined metric that incorporates multiple individual measures to provide a single score) are preferred to: *i)* aggregate the input and output data into a single comprehensive measure of performance; *ii)* determine if the critical aspects of service delivery have been achieved.

Traditionally, program measures have been evaluated against absolute standards estimated as global average values, mainly focusing on controllable input variables such as staff and capital. The analysis may be based on ‘best performance frontier’ and/or ‘central tendency (average-based’) techniques, although the two perspectives can potentially result into different improvement decisions.[21] Furthermore, there exists variants of either of the “frontier” methods and regression analyses. Whether to prefer either one or combination of the methods depends on the study context and objectives, data characteristics and user skills.

The frontier methods include non-parametric data envelopment analysis (DEA) and parametric stochastic frontier analysis (SFA). Both can be used to identify a production frontier for a group of facilities but they employ different assumptions and methodologies. DEA methods use mathematical programing to obtain the production frontier enveloping all the observed data. Specifically DEA estimates efficiency scores for each unit by comparing its input mix (normally the resources necessary to complete a task) and volume of services provided against the best performing peers in the set. In models assuming variable returns to scale unit comparison is restricted to only among those with comparable sizes. The scores obtained depend on model characteristics and level of input variables used by best performing facilities in terms of their outputs to inputs ratio. They reflect the performance of each facility relative to best performing ones. The exact interpretation depends on the DEA model orientation used, whether output-maximizing or input-minimizing. Limitations of DEA include sensitivity to outliers, assumes no errors (which may bias results) and standard models do not permit hypothesis testing for the best model specification.[22]

Conversely, stochastic frontier methods are parametric. Typically they accommodate only a single input with multiple outputs; can differentiate errors from inefficiency sources; require specification of a functional form and; permit computation of the confidence intervals for efficiency scores and their best predictors for individual facilities. However, based on parameter estimates it may not envelop all output points and does not identify peers.[22] Regarding regression methods, least squares are used to define functional relationships between one dependent variable and other or multiple independent ones and to predict sources of variations. The methods estimate a single sample-based global average score and is amenable to hypothesis testing.

Increasingly, data envelopment analysis (DEA) is becoming instrumental in evaluating health service delivery efficiency, which is typically complex and multidimensional. Preference for DEA accrues from: *(i)* its capacity to integrate multiple input and output data of any measurement (both controllable and those beyond a provider’s control) and dimension simultaneously [21] to produce a single aggregate relative “efficiency score” for each service unit. These scores, adjusted to be a number between 0 and 1 (0–100%) are relative measures estimated based on the most favorable combination mix for each unit in contrast to using an absolute standard; *(ii)* ability to construct a ‘best practice’ frontier and simultaneously compare facilities to classify each unit most favorably; *(iii)* no need for inclusion of cost variable nor modelling of functional relationships for inputs to outputs;[23] *(iv)* ability to identify respective unit productivity individually, sources of inefficiencies as well as the benchmark peers (‘peers’ = units assigned a score of 100%) in the set plus their respective weight to guide improvements required for the less efficient ones. However, it does not reveal how to accomplish the needed changes. Ideally, the improvement efforts prioritized by a manager for respective facilities should consider their practicality and feasibility.[24,25]

In the current study, since efficient resource use and output maximization are the key objectives of the VMMC serviced delivery, DEA-based output-orientated technical efficiency and Malmquist productivity index (MPI) are used respectively to: *i)* demonstrate extent of resource use by facilities to maximize VMMC service outputs; *ii)* measure total factor productivity change and identify sources of variation by estimating technical efficiency change and efficiency change between 2011(low season and routine services) and 2012 (period of accelerated VMMC activities). Malmquist Productivity Index (MPI) is interpreted as a measures of total factor productivity change over time and its components (efficiency change and technology change) provide insight into the sources of observed variations in VMMC target outputs. Essentially, it distinguishes productivity changes that are due to increased efficiency (catching-up with best-practice facilities) from technological changes, e.g. service delivery strategies/techniques adopted. The efficiency change component is a product of scale and pure efficiency and shows the position of a facility relative to the frontier made up by “best practice” units. The technical change component measures how much the frontier shifts relative to comparable units. In either case the index values greater, equal to, or less than one indicate improvement, stagnation or regress. Since MPI values are percentiles, they are expressed as geometric means. Its key benefit is that it does not require information on the prices of inputs and outputs. Furthermore, calculation of this index requires no assumptions regarding orientation of the organizations under analysis.[26,27]

The strategic importance of using DEA techniques to evaluate efficiency of medical male circumcision services is that multiple dimensions are assessed simultaneously; each unit is ranked according to the most favorable performance relative to similar ones in the set; DEA-estimated frontier is a good approximation of the true underlying production possibilities; it provides guidelines for objective benchmarking and setting production objectives for less efficient units; and it enables productivity evaluation of performance over time. Whereas the DEA outputs provide diagnostic performance information for a set of comparable service delivery units, it is desirable that management decisions further consider broader policy objectives such as service access and coverage as well as prevailing exogenous factors.

### Considerations for constructing DEA model

**Variable selection**. Although any set of variables may be chosen for the DEA model, the outputs preferred for the current study closely reflect the organizational context plus their functional relationships.[27,28,29,30,31] Using an arbitrary approach inherently may exclude important performance variables [27,30]. Variables incorporated included clinicians, nurses, surgical beds and total elapsed circumcision time [32,33] as inputs while number of circumcisions, proportions of circumcised receiving HIV tests and quality dimensions [34] were outputs. The quality variable was constructed using principal component analysis (PCA) and exploratory factor analysis techniques. Fifteen process items that correlated highly (conventionally set at ≥0.4) with factor 1 were identified as the critical quality measure items, thus were used to construct composite index for scoring service quality per facility. Final index scores were obtained by averaging scores across items assessed, with higher coefficient scores representing higher quality on a percentile scale [34].

**Model orientation**. Clarifying model orientation provides information on how efficiency scores are derived and how they vary. Technical efficiency indicators may be either input-oriented or output-oriented depending on which variable set the program managers have control. Input-orientated technical efficiency focuses on minimizing inputs used without reducing the output quantity while in output-orientated efficiency the focus is on expanding output quantities while maintaining current level of inputs. Technical efficiency (global TE) is a product of pure technical (PTE) and scale efficiency (SE). Pure TE is generally associated with organization of operations of the specific service producing units (input-output mix) while scale depicts issues related to size and indicates if the facility is too large or too small considering the inputs used to produce the observed outputs. Sources of inefficiency of a facility unit may thus be attributable to either or both of the components [35]. Scale efficiency of a service delivery unit may be examined under different model versions which make different assumptions about returns to scale: constant returns to scale (CRS), variable returns to scale (VRS) and non-increasing returns to scale (NIRS).[36] The scale efficiency (SE) is given by the ratio between the CRS and VRS technical efficiency scores.[23,37,38] Scale inefficiency (SE<100%) may occur if the facility is not operating at its most productive/optimal size (in terms of its output-input mix), due to: *i)* increasing returns to scale (TE_VRS_ > TE_CRS_); or *ii)* decreasing returns to scale (TE_VRS_ = TE_NIRS_).[27,39] The VRS model, allows the best practice level of outputs to inputs to vary with the size of the facilities assessed whereas under CRS it is determined by the highest achievable ratio of outputs to inputs for each unit, regardless of size. Efficiency scores are identical when computed using input or output orientation under CRS but may vary under VRS. Also, scores obtained when assuming VRS may be higher than or equal to CRS ones since they indicate only technical inefficiency resulting from non-scale factors.[40,41].

## Materials and Methods

### Ethics statement

The project was approved by the Ethics Review Committee of the Kenya Medical Research Institute. Written informed consent was obtained from each participant in the study. Academic approval was obtained from Maseno University.

### Study design and setting

Using a comparative process evaluation of voluntary medical male circumcision (VMMC) scale-up in Nyanza, site level data was collected among randomly sampled facilities providing VMMC services as fixed, out-reach and mobile sites (15/12/3) during two rounds of Systematic Monitoring of Medical Male Circumcision Scale-up (SYMMACS) in 2011 and 2012.[9] The first round was conducted during low season while round two occurred during peak season with accelerated activities. Assessment of service tasks performed, availability of guidelines, supplies and equipment and, continuity of care was conducted using modified national VMMC monitoring instruments.

### Sample size for DEA

Of all facilities observed, only 9 fixed and 12 outreach VMMC facilities meeting the model requirements were included in the current study. The following recommendations regarding sample size requirements for performing DEA were considered from literature:[42,43]

- It should be larger than the product of the number of inputs and outputs;
- It should be at least 3 times the sum of the number of inputs and outputs.

Given 4 input and 3 output variables, the minimum sample size would be at least 12 (4X3) based on # (*i*) or 21 [3(4+3)] based on #(*ii*).[42] Given these considerations, 21 VMMC facilities was considered a sufficient sample.

### Choice of model input-output variables

Hacer and Ozcan [44] recommend multiple outputs specification instead of one to reduce measurement error occasioned by varied input requirements, although effects of within-group homogeneity or between-group heterogeneity should be similarly considered. Bessent and Bessent [45] have proposed a criteria for identifying relevant input and output variables to ensure DEA performance remains robust [43]:

1. A realistic relationship of inputs to outputs.
2. Functional relationship of measured inputs to outputs.
3. The relationship is such that increases in inputs are associated with increases in outputs.
4. The measurements have no zero elements, but where measurements which have legitimate zero values exist, a small value (.01) is added to satisfy the model requirement.

The model variables considered for this DEA are summarized in Table 1.

**Table 1:** Table showing model input and output variables and their definitions.

Index	Definition of unit of measurement
**Inputs**	
Clinician	Number of clinicians staff performing MC
Nurse	Number of nursing staff performing MC
Surgical beds	Number of surgical beds in use in the MC theatre
Total operating time (min/sec)	Total elapsed client-surgeon contact time during circumcision
**Outputs**	
MCs performed	Number of procedures performed by clinician/nurse
HTC performed (%)	Proportion of pre-surgical HTC
Quality of service	Average facility service quality index score (quintiles)

Nurses and clinical officers are trained to perform the procedure as either primary or secondary providers hence a facility may have a mix of both or only one of the cadres. There were no medical doctors in the sample. Selected variables have programmatic relevance or functional relationships.

### Data analysis

Data envelopment analysis was performed using PIM DEAsoft Ver 3.2, by Ali Emrouznejad and Emmanuel Thennassoulis (2010). Paired t-test was performed to compare means of the obtained efficiency score and *Mann Whitney U* test to compare productivity scores using *SAS v*. *13* software.

### Rationale for the performance model

In performing DEA selecting an appropriate variable set and specifying model specification and orientation is a methodological necessity to ensure results are comprehensive and robust. Variables included in the DEA model (Table 1) were considered most critical to circumcision process.[46] The number of circumcisions, surgical beds in use and uptake of pre-operative HTC were considered to be outside the control of providers (exogenous factors) since they depend on demand for VMMC. Consequently, the maximum possible increase in outputs by facility was estimated while keeping the inputs and exogenously fixed outputs at their current levels. As demonstrated by Banker and Morey [47] this consideration allows non-discretionary variables to influence the relationship between inputs and outputs, but the “efficiency score” is not affected by them (since they are considered fixed and out of the control of the providers). It also improves comparability of units in the set and enhances opportunities for identifying target increases in the controllable variables required for the facilities to be efficient.

### Model orientation

We assumed an output orientation with variable returns to scale since the program aims to maximize outputs within constrained resources. VMMC facility size (in terms of number of clinical staff and beds used) was deemed relevant to assessing relative efficiency. At the same time, bed space, number of staff, uncertain service demand and other exogenous constraints were likely to cause VMMC facilities to operate at suboptimal capacities.[2] In these circumstances, the VRS assumption ensures that a facility is only compared against others with similar size (based on number of staff and beds).[23,42] Other model versions were computed to elicit the marginal productivity of service units under different assumptions. The efficiency scores obtained indicate extent of input use for the maximum possible outputs obtained with given unit sizes.[42]

### Malmquist productivity index (MPI)

Productivity index measures how output changes with the level of inputs used between two time-intervals *(t*, *t+1)*. Values indicate shifts in productivity for each production unit relative to (towards, along or away from) the observed frontier.[27] Index values >1 implies productivity growth, while a value <1 shows productivity decline, and if = 1 indicates stagnation. Thus production quantities and technological best practice can be shown to be improving, deteriorating or unchanging over time. Malmquist productivity index is one of the methods commonly used to assess productivity changes over time. It identifies sources of productivity change in terms of: *i)* technical change (associated with variations in quantity and quality of labor/capital, for example clinical staff skills by cadre and bed space); *ii)* pure efficiency change (associated with variations in context/organizational approach largely of labor and capital inputs, including compliance with VMMC treatment protocols and referrals, support supervision, availability of supplies). Both i & ii constitute the overall efficiency change and; *iii)* scale efficiency (which measures productivity changes attributable to variation in unit size, for example staff mix and work responsibilities, work space and logistics). If there is improved use of resources the service unit position will move towards the frontier indicating positive efficiency gain.[48] The Malmquist productivity index was estimated based on Ray and Desli (1997) method in Cooper *et al*., 2007 [21] to account for scale efficiency change effects as the output mix varied over time with changes in the number of staff and surgical beds used.[49] The average efficiency changes between the two time-periods considered are represented by geometric means to normalize values because multiple items with different properties are involved.

### Weighting considerations

No *a priori* weight restrictions were imposed on the variables.

### Identification of peers

Based on model specifications with exogenous factors fixed, conventional DEA efficiency evaluation of VMMC facilities was performed simultaneously and a reference set of efficient units (peers) identified using a two stage process to ensure identification of both high quality-high efficiency peers. The procedure also identified potential changes required to make each inefficient unit as efficient as the most efficient (best-practice) ones on the frontier [23].

## Results

### Technical efficiency scores

**Input variables**. During 2011, each facility had a mean of 1.5 clinicians and nurses respectively. Mean number of surgical beds reduced from 1.8 in 2011 to 1.2 in 2012 while mean total elapsed operation time improved significantly from 32.8 minutes (SD 8.8) in 2011 to 30 minutes (SD 6.6) in 2012 (95%CI = .0350–5.2488; *t =* 2.114; *df*. = 20; *p =* 0.047).

**Output variables**. The mean number of circumcisions performed increased from 894.4 (SD 903.4) in 2011 to 1066.6 (659.) in 2012. Mean quality index score in 2011 was at 49.7^th^ percentile compared to 53^rd^ percentile in 2012, but proportion of pre-surgical HTC declined from 75.6% (SD 20.6) to 70.9% (SD 27.8) (Table 2).

**Table 2:** Facility actual production inputs and outputs by year based on output oriented VRS DEA model.

2011								2012						
Facility	Clinician	Nurse	Surgical beds	Operation time (min/sec)	MCs performed	HTCs % performed	Quality Score*	clinician	Nurse	Surgical beds	Operation time min/sec	MCs performed	% HTCs performed	Quality Score*
103	1	2	2	50.5	1325	7	1	1	1	1	31.29	1614	86.2	85
123	4	3	3	40.05	2488	83.2	60	1	1	1	36.57	1947	85.1	60
118	1	1	2	29	1000	68.8	80	0	2	2	28.75	837	91.7	30
102	1	1	2	34.57	137	58	90	2	0	1	21.38	803	77.1	70
125	0	1	1	29.39	414	75	80	2	1	1	28.41	1438	71.7	85
126	1	1	4	24.14	256	98	85	1	1	1	35.01	691	83.8	60
108	1	1	3	28.15	785	87.5	40	1	1	1	32.53	376	65	30
107	1	1	1	42.2	242	70.8	80	2	0	1	30.22	1003	62.9	85
119	1	1	1	28.43	887	84.3	92	1	1	1	30.56	764	97.1	25
104	1	1	2	34.57	137	58	40	1	1	2	18.17	739	79.8	50
111	1	1	4	24.14	256	98	55	2	0	1	16.05	828	66.4	85
110	4	3	3	40.05	2488	83.2	10	2	0	1	20.36	63	84.8	50
112	3	2	4	34.3	1136	86.9	10	1	2	1	30.28	1430	70.3	85
129	1	1	1	23.34	176	73	50	0	2	1	24.19	219	77	15
124	1	2	1	36.11	516	90	80	1	2	2	50.5	1325	7	1
101	3	3	1	32.54	2718	66	1	1	1	1	23.1	2897	97.6	85
130	1	1	1	39.46	23	80	5	1	1	1	25.19	1507	99.3	30
105	1	1	1	23.46	342	74	30	0	2	1	49.32	552	78.7	15
121	0	2	1	46.36	30	0.01	90	0	2	2	35.42	1516	0.01	40
114	3	2	4	23.39	2850	99.1	50	1	1	2	29.13	1616	85	60
109	1	1	1	29.26	688	71	15	2	1	1	25.06	243	49.9	70
**Mean**	**1.5**	**1.5**	**2.0**	**33.0**	**899.7**	**72.0**	**49.7**	**1.1**	**1.1**	**1.2**	**29.6**	**1067.0**	**72.2**	**53.1**
**SD**	**1.17**	**0.75**	**1.20**	**7.90**	**938.82**	**25.69**	**32.99**	**0.70**	**0.70**	**0.44**	**8.75**	**675.59**	**25.96**	**27.40**

Few sites had either zero-clinician or nurse as tasks could be performed by either cadre. In the model, the zeroes were substituted with 0.01, since the model does not work with zero. The number of VMMC providers for each site was based on those available on days of data collection visit.

*Quality score in quintiles; no circumcisions conducted in facility 106 in 2011.

**Efficiency scores**. The average technical efficiency scores under CRS, VRS and Scale models were 76% (SD 28.7); 84% (SD 25.3) and 91% (SD 19.8) in 2011 compared to 89% (SD 25.2); 89% (SD 25.1) and 99% (SD 4) in 2012. The increase in scale technical efficiency was statistically significant (95%CI-31.47959–4.698508; *t =* -2.8179; *df*. = 20; *p =* 0.005) but not for CRS and VRS. Thirteen facilities (61.9%) scored 100% (TE_VRS_) in 2011 compared to 16 (76.2%) in 2012 (Table 3).

**Table 3:** Output oriented Technical Efficiency Scores of facilities by year, type, and return to scale (n = 21).

Facility type and #		CRS efficiency		VRS efficiency		NIRS Efficiency		Scale efficiency	
		2011	2012	2011	2012	2011	2012	2011	2012
Type A—fixed facilities	Fac. 103	75	100	100	100	75	100	75	100
	Fac. 123	57	100	100	100	100	100	57	100
	Fac. 118	82	100	91	100	91	100	90	100
	Fac. 102	100	100	100	100	100	100	100	100
	Fac. 125	100	100	100	100	100	100	100	100
	Fac. 126	92	71	92	71	92	71	100	100
	Fac. 108	43	35	43	35	43	35	100	100
	Fac. 107	97	100	100	100	97	100	97	100
	Fac. 119	100	100	100	100	100	100	100	100
Type B—Outreach facilities	Fac. 104	44	100	44	100	44	100	100	100
	Fac. 111	100	100	100	100	100	100	100	100
	Fac. 110	18	100	100	100	18	100	18	100
	Fac. 112	100	100	100	100	100	100	100	100
	Fac. 129	100	100	100	100	100	100	100	100
	Fac. 124	89	1	89	1	89	1	100	81
	Fac. 101	100	100	100	100	100	100	100	100
	Fac. 130	100	100	100	100	100	100	100	100
	Fac. 105	33	100	33	100	33	100	100	100
	Fac. 121	100	100	100	100	100	100	100	100
	Fac. 114	56	71	56	71	56	71	100	100
	Fac. 109	19	82	25	82	19	82	76	100
**Summary statistics**	**Mean**	**76**	**89**	**84**	**89**	**79**	**89**	**91**	**99**
	**SD**	**28.7**	**25.2**	**25.3**	**25.1**	**28.9**	**25.1**	**19.8**	**4.0**

Table shows the level of resource utilization based on the technical efficiency scores by constant, variable return to scale and Non-increasing Return to Scale; Scale efficiency is a ratio of the CRS: VRS. A difference between the efficiency values under NIRS, VRS and CRS indicate scale inefficiency exists. For example, if TE_CRS_<TE_VRS_; TE_VRS_>TE_NIRS_, size is too small, while TE_NIRS_ = TE_VRS_ indicate size too big and at optimal size if TE_VRS_ = TE_CRS_. Additionally, relatively higher standard deviations reflect wider dispersals from the ‘best practice’ units.

**Efficiency scores by service delivery type**. The mean technical efficiency (TE_VRS_) increased significantly among outreach facilities from 78% (SD 29.4) in 2011 and 84% (SD 30.9) in 2012. Likewise, the outreach facilities also improved significantly in scale technical efficiency change (95%CI-45.08035–2.547979; *df*. = 11; *t =* -2.4647; *p =* 0.015). The decline in TE_VRS_ from 92% (SD 19.6) in 2011 to 91% (SD 14.5) in 2012 among fixed facilities was not statistically significant. (Table 4).

**Table 4:** Summary of facility performance scores by type and year.

Facility	Statistic	CRS Efficiency		VRS Efficiency		Scale Efficiency	
		2011	2012	2011	2012	2011	2012
Type A—fixed facilities	Mean (%)	83	90	92	90	91	100.0
	SD	21	23	19	23	14.5	0.0
	#100% TE (n = 9)	3	7	6	7	5	9
Type B—Outreach facilities	Mean (%)	77	84	78	84	97	98
	SD	30.7	30.9	29.4	30.9	7.6	5.9
	#100% TE (n = 12)	6	9	7	9	10	11

SD = standard deviation; TE = technical efficiency; CRS = constant return to scale; VRS = variable return to scale. TE_VRS_ significantly increased among outreach facilities.

### Identification of ‘best practice’ peers and benchmarking

Table 5 shows an output oriented VRS results of inefficient facilities and their peers with respective combination weights in parenthesis. These show projected production options that will enable them reach relative efficiency. Facilities identified as peers were #111, 119, 121, 129 and 125 in 2011; 103 and 101 in 2012. The reference facilities #129 and 111 had high technical efficiency scores but low in quality score (50 and 55 respectively). We repeated the DEA model excluding the 2 low-quality units to enhance probability of obtaining only high efficiency-high quality peers, following Sherman and Zhu (2006)[50] and, Shimshak, Lenard and Klimberg (2009).[51]. The resulting new reference units shown in Table 6 were obtained. All had higher efficiency scores and quality values. Facilities 119 and 125 dominated in 2011 and 103 in 2012.

**Table 5:** Initial DEA results showing inefficient units, their corresponding efficiency reference sets and relative weight respectively assigned to each.

DMU	Efficiency score	Reference facility (relative weights)
**2011**		
108	43%	Fac. 119 (1)
126	92%	Fac. 119 (1)
109	25%	Fac. 129 (0.78); Fac. 121 (0.22)
105	33%	Fac. 121(1)
114	56%	Fac. 121(1)
118	91%	Fac. 125(0.32); Fac. 119 (0.68)
104	44%	Fac. 121 (1)
124	89%	Fac. 111 (1); Fac. 129 (0.84)
**2012**		
126	71%	Fac. 103 (1)
108	35%	Fac. 103 (1)
112	112%	Fac. 101 (1)
124	1%	Fac. 101 (1)
114	71%	Fac. 101 (1)
109	82%	Fac. 101 (1)

DMU = Decision-making unit; fac. = facility; Peer count/freq = number of times the facility is a peer (in 2011, Fac. # 119 appear 3 times; #121 also 3 times; #129 twice while #125 and 111 each appear once. In 2012, Faac #103 appeared twice and #101 appeared four times. Appropriate combination weights (λ) required to enable respective facilities reach relative efficiency are in parenthesis.

**Table 6:** Revised DEA model results after deleting low quality facilities: inefficient units, their corresponding efficiency reference sets and relative weight respectively assigned to each.

DMU	Efficiency score	Reference facility(relative weights)
**2011**		
118	91%	Fac. 119 (0.68); Fac. 125 (0.32)
108	43%	Fac. 119 (1);
126	92%	Fac. 119 (1)
**2012**		
126	71%	Fac. 103 (1);
108	35%	Fac. 103 (1)

DMU = Decision-making unit; fac. = facility; peer count/frequency count is the number of times the facility appear as a peer (thrice for fac. #119 in 2011 and twice for fac. #103; figures in parenthesis are appropriate combination weights required to enable respective facilities attain efficiency.

### Productivity measures: factors associated with productivity change

**Productivity performance by Malmquist Index**. There was significant progress in observed overall total factor productivity of 83.4% (*p = 0*.*032*) as well as in technical change of 72.9% (*p =* 0.008). Pure efficiency change progressed by 21% and scale efficiency change by 6.3% (Table 6). Facilities #103, 123, 118, 104, 107, 110, 111, 112, 129, 130, 105, 114 & 109 experienced progress in total productivity growth and #108, 124 & 126 regressed (Table 7).

**Table 7:** Productivity performance for each service delivery facility by type.

Facility code	TC	SEC	PEC	TFPG (MI)	Efficiency-2011	Efficiency 2012
Fac. 103	1	1.16	1	1.16	100	100
Fac. 123	1	1.33	1	1.33	100	100
Fac. 118	0.96	1.18	1.1	1.24	90.75	100
Fac. 102	1	1	1	1	100	100
Fac. 125	1	1	1	1	100	100
Fac. 126	0.93	1	0.76	0.71	92.39	70.59
Fac. 108	0.63	1	0.81	0.51	43.48	35.29
Fac. 107	1	1.02	1	1.02	100	100
Fac. 119	1	1	1	1	100	100
Fac. 104	0.97	1	2.25	2.19	44.44	100
Fac. 111	1.14	1	1	1.14	100	100
Fac. 110	2.92	2.34	1	6.83	100	100
Fac. 112	2.92	1	1	2.92	100	100
Fac. 129	1.3	1	1	1.3	100	100
Fac. 124	8.96	0.22	0.01	0.03	88.89	1.18
Fac. 101	1	1	1	1	100	100
Fac. 130	4.12	1	1	4.12	100	100
Fac. 105	0.97	1	3	2.92	33.33	100
Fac. 121	1	1	1	1	100	100
Fac. 114	1.16	1	1.27	1.47	55.56	70.59
Fac. 109	1.32	1.08	3.23	4.62	25.49	82.35
**Geometric mean**	**1.729**	**1.063**	**1.211**	**1.834**	**84.492**	**88.571**
**SD**	**1.822**	**0.345**	**0.716**	**1.586**	**25.326**	**25.120**

Since Malmquist indices are percentile quantities, the geometric mean is used whenever averaging is carried out. Values greater (smaller) than one shows productivity improvement (decline). TC: technical change; SEC: scale efficiency change; PEC: pure efficiency change; [efficiency change = SEC+PEC and demonstrates modest progress towards best practice frontier between 2011 and 2012]; TFPG (MI): total factor productivity growth (Malmquist Index). While, individual productivity values vary widely, mean TPFG significantly improved by 83.4%. The key driver of technical efficiency was technical change (72%) largely attributable to outreach service delivery and improved total elapsed surgical time.

Significantly more (10/12) outreach facilities progressed in total factor productivity (TPFG) (*p =* 0.032) and (9/12) technological change (TC) (*p =* 0.008) compared to the fixed ones, which experienced a decline in total factor productivity growth (TPFG) and technological change (TC) (0.3% / 5.3%). The observed decline in pure efficiency change (PEC) by 3.7% and progress in scale efficiency by 2.3% among fixed facilities were not significantly different than the respective changes among the outreach ones (Table 8).

**Table 8:** Productivity indices for VMMC facilities by types between 2011 and 2012.

Facility type/Statistic		TC	SEC	PEC	TFPG	2011 (TE %)	2012 (TE %)
**Type ‘A’ (fixed)**	Mean	0.947	1.077	0.963	0.997	91.847	89.542
	SD	0.114	0.113	0.101	0.240	17.447	21.260
**Type ‘B’ (outreach)**	Mean	2.315	1.053	1.397	2.462	78.976	87.843
	SD	2.235	0.445	0.899	1.854	28.690	27.641

TC: Technological change (Boundary shift due to technological change); SEC: Scale Efficiency Change; PEC: Pure technical Efficiency Change; TFPG: Total Factor Productivity Growth (Malmquist Index); Values equal to 1 implies no productivity change; <1 decline and, >1 progress in productivity. TE: technical efficiency score (%).

## Discussion

### Technical efficiency

The observed technical efficiency results suggest that, given the quantity of inputs they consumed, the facilities could have produced 16% more output in 2011 and 11% more output in 2012. The observed distribution of technical efficiency scores under VRS (which expresses only pure technical inefficiency excluding scale factors, and is associated with managerial factors) and CRS, (which expresses global technical inefficiency comprising both pure and scale efficiency) shows that on average facilities exhibited mainly scale inefficiency in 2011. The significant improvement in scale technical efficiency scores during 2012 shows the facilities were able to use the resources more favorably to increase output indicating a positive impact of accelerated activities. The relative unit sizes improved, in terms of the number of input resources used by facilities vis-à-vis outputs produced. The significant improvement in total elapsed operation time (time-saving effect) likely enabled teams to improve their capacity to produce more with the same (less) input, thereby exploiting (minimizing) their unused potentials. Rech *et al*., (2014) in a survey of VMMC program in South Africa observed that the reduced operation time was not associated with poor service quality.

On the other hand, the pure technical inefficiency elicited under VRS could be related to largely unsatisfactory performance of tasks including compliance to standard guidelines for service delivery. Additionally, among fixed facilities, observed inefficiency could be associated with dynamic contexts, inelastic obligatory institutional requirements and personnel factors that adversely affect technical efficiency.[9,52] In previous DEA evaluation of health care delivery at various delivery tiers in Kenya Kirigia and colleagues [29,31] demonstrated that technical inefficiencies was largely associated with unexploited resources. The present study similarly highlights the critical importance of resource use in VMMC service delivery. Hence, it is recommended that program supervisors should include management solutions in planning their routine operations.

Overall, DEA technique is particularly a useful tool to use for first-line evaluation to furnish vital diagnostic information on VMMC facility performances. However, since it cannot generally identify the ‘causes’ of inefficiency with the precision a manager would need in order to take decisive action, additional investigation on the improvement needs identified using other methodologies is necessary. [53]

### Benchmarking

In DEA-based benchmarking, respective unit performance is assessed against the efficient frontier or best practice units in the sample as opposed to an ‘average’ or ‘central tendency’ analysis. In the current study, the benchmark facilities were all of fixed facility category. This could be attributed to unique and diverse experiences among outreach service categories in terms of size and operational dynamics. This implies that when planning improvement efforts, based on benchmarking, it is necessary for managers to consider the contextual needs of facilities and other occult causes of inefficiencies unique to them despite their position relative to the frontier.

Stratifying facilities using multiple criteria in a step wise approach [54] improves the precision of DEA benchmarking exercise as observed in this study. Inclusion of service quality variable in DEA benchmarking in the current study enhanced evaluation comprehensiveness and balance, similar to previous studies [51,55]. However, Sherman and Zhu (2006) have observed an efficiency/quality trade-off when benchmarking with quality-adjusted DEA to seek lower-cost-high quality service in the banking industry. Shimshak *et al*., (2008) recommend that “the choice of quality output measures be appropriately related to the input measures”[51] to improve compatibility with the objectives of the DEA model.

### Productivity measures and sources of variation in VMMC service delivery

The present study has used DEA to identify the scope of technical inefficiency, insofar as they are pure inefficiency (context / organizational-related), technology-related, or scale-related. The main driver of productivity increase was technical change largely related to accelerated program activities in 2012 that enabled facilities to expand their production possibilities. For example, improved ‘speed’/experience in performing circumcisions enabled providers to perform more procedures without additional staff/bed space as inputs. The significant progress in technical efficiency change especially related to pure efficiency change among outreach facilities suggest the majority were versatile and aptly exploited their production resources/possibilities to expand productivity, hence importance of flexible program strategies. However, the modest progress in scale efficiency change indicates that facility size was not a major source of the improved productivity observed. In 2011, the majority of facilities did not exhibit optimal productive unit size.

The decline in factor productivity among fixed VMMC facilities was attributable mainly to regress in technical change, technical efficiency change and pure efficiency change which reflect probable influence of operating environments, staff skills and other institutional management factors. These facilities face challenges to optimally adjust to variations in service demands due to inelasticity in obligatory resources, especially related to personnel issues, supplies and theatre-space.[56] Consequently the Ministry of Health policies and implementing organizations could seek to emphasize improvements of operational contexts of fixed facilities through strategic resource allocation and investment in staff skills. This is more critical when considering mainstreaming VMMC for long-term sustainability. However, outreach service delivery model remains strategic for efficient resource use.[38,57]

### Study limitations

Since DEA technical efficiency scores exhibit unknown statistical distribution and that the efficiency scores by CRS, VRS and Scale may be skewed the statistical inferences should be interpreted with caution. DEA assumes all errors are due to inefficiency and its estimates are sensitive to outliers.
